# Dacryoadénite sarcoïdosique

**DOI:** 10.11604/pamj.2014.18.313.4219

**Published:** 2014-08-21

**Authors:** Rajae Derrar, Wafae Cherkaoui

**Affiliations:** 1 Service d'Ophtalmologie A, Hôpital des Spécialités, CHU de Rabat, Maroc

**Keywords:** Dacryoadénite sarcoïdosique, hypertrophie, glandes lacrymales, Sarcoid dacryoadenitis, hypertrophy, lacrimal glands

## Image en medicine

Patient âgé de 40 ans présentant depuis 2 mois une hypertrophie bilatérale des glandes lacrymales avec photophobie et larmoiement. L'examen trouve une acuité visuelle à 10/10 au niveau de l'oeil droit et gauche, une sécheresse oculaire avec un examen à la lampe à fente qui montre un segment antérieur et postérieur normaux. L'examen général est normal. Une sarcoïdose a été suspecté, un bilan a été demandé qui comprend une radiographie pulmonaire, une recherche de l'enzyme de conversion d'angiotensine ainsi qu'une biopsie de la glande lacrymale. L'Enzyme de conversion d'angiotensine était élevée, la biopsie a montré des granulomes épithélioïdes gigantocellulaires sans nécrose caséeuse. Une corticothérapie systémique a été débutée, les glandes lacrymales ont retrouvé leur taille normale au bout de 3 mois de traitement. La sarcoïdose est une affection granulomateuse généralisée chronique de cause inconnue dont la symptomatologie inaugurale peut être ophtalmologique.

**Figure 1:**
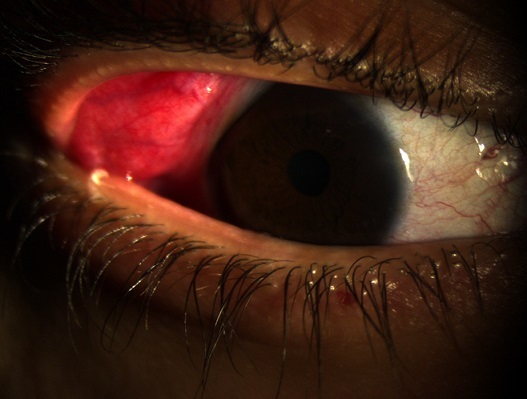
Hypertrophie de la glande lacrymale

